# Heuristics for the inversion median problem

**DOI:** 10.1186/1471-2105-11-S1-S30

**Published:** 2010-01-18

**Authors:** Vaibhav Rajan, Andrew Wei Xu, Yu Lin, Krister M Swenson, Bernard ME Moret

**Affiliations:** 1 Laboratory for Computational Biology and Bioinformatics, EPFL, CH-1015 Lausanne, Switzerland

## Abstract

**Background:**

The study of genome rearrangements has become a mainstay of phylogenetics and comparative genomics. Fundamental in such a study is the median problem: given three genomes find a fourth that minimizes the sum of the evolutionary distances between itself and the given three. Many exact algorithms and heuristics have been developed for the inversion median problem, of which the best known is MGR.

**Results:**

We present a unifying framework for median heuristics, which enables us to clarify existing strategies and to place them in a partial ordering. Analysis of this framework leads to a new insight: the best strategies continue to refer to the input data rather than reducing the problem to smaller instances. Using this insight, we develop a new heuristic for inversion medians that uses input data to the end of its computation and leverages our previous work with DCJ medians. Finally, we present the results of extensive experimentation showing that our new heuristic outperforms all others in accuracy and, especially, in running time: the heuristic typically returns solutions within 1% of optimal and runs in seconds to minutes even on genomes with 25'000 genes--in contrast, MGR can take days on instances of 200 genes and cannot be used beyond 1'000 genes.

**Conclusion:**

Finding good rearrangement medians, in particular inversion medians, had long been regarded as the computational bottleneck in whole-genome studies. Our new heuristic for inversion medians, ASM, which dominates all others in our framework, puts that issue to rest by providing near-optimal solutions within seconds to minutes on even the largest genomes.

## Background

### Introduction

The advent of high-throughput sequencing, combined with the development of automated annotation tools (such as gene hunters), has created entirely new fields of application for computational methods in biology, most notably comparative genomics. Comparing two or more genomes requires models of evolution at various scales, from the well established sequence evolution models to more speculative models of structural changes, such as rearrangements, duplications, and losses. Genomic rearrangements are evolutionary events through which regions of the genome are moved around, but without any alteration of sequence contents; simple examples include transposition (moving a part of the genome to another location) and inversion (reversing in place a part of the genome). The study of genome rearrangements dates back to the pioneering days of genetics: Sturtevant and Dobzhansky identified inversions in the genome of *D. melanogaster *and even used these inversions as characters in a phylogenetic analysis [1,2]; further work (on plants) came out of Palmer's laboratory nearly 50 years later [3]. However, it was the advent of whole-genome sequencing that provided the main impetus--as manual analysis of a few inversions had to give way to computational analyses of hundreds or thousands of rearrangements.

Sankoff [4] first identified two formal questions about rearrangements: (i) given two genomes, what is their edit distance, that is, the length of the shortest series of rearrangements that can transform one into the other and (ii) given three genomes, what is a median, that is, a fourth genome that minimizes the sum of the pairwise edit distances between itself and the other three. These problems have by now a large literature [5]. While the edit distance can be computed in linear time [6,7], the median problem is known to be NP-hard for most formulations (see [8,9] for surveys of these formulations). While exact median solvers have been used, most notably in GRAPPA [10], their computational requirements have limited their use to very small genomes, such as those of organelles (see, e.g., [11]). In order to work on larger genomes, a large number of heuristics have been proposed over the years. Most of these heuristics operate through a type of greedy search by searching for a "good" move to make, making that move, and iterating until no further good move can be found.

In this paper, we provide a formal framework for this type of iterative greedy heuristic; this framework allows us to classify, characterize, and gain insight into proposed heuristics. In turn, these insights enable us to devise a general principle for such heuristics: that the best continue to base their computations on the original input data for as long as possible (as opposed to using new values computed in previous iterations). Using this principle, we devise an entirely new median heuristic by leveraging an exact solver for DCJ medians developed by one of us. Finally, we present the results of extensive experimentation with existing median heuristics and our new heuristic, showing that our new heuristic is both more accurate and much faster than existing ones, to the point where even the largest genomes (with tens of thousands of genes) can be handled very quickly.

### Terminology, notation, and definitions

We assume that inversion is the only evolutionary event; since inversions do not alter gene content, we further assume that all genomes under consideration have the same set of genes. Finally, since inversions cannot move genomic regions between chromosomes, we assume that the the genome is viewed as a single permutation. While these assumptions are clearly unrealistic when phrased in terms of genes (somewhat less so when phrased in terms of syntenic blocks), they are used in most of the published work.

A genome consisting of *n *genes is represented by a signed linear permutation on the elements {1, ..., *n*}; if that permutation is *π*, we write the ith element of the permutation as *π*_*i*_. An *inversion*, *ρ *(*i*, *j*) on a permutation *π *= (*π*_1 _... *π*_*i*_... *π*_*j*_... *π*_*n*_) reverses all the elements between (and including) *π*_*i *_and *π*_*j *_while changing their signs, yielding *π*. *ρ*(*i*, *j*) = (*π*_1_... *π*_*i*-1_-*π*_*j *_... -*π*_*i*_*π*_*j*+1_... *π*_*n*_). For signed permutations A and B, the *inversion distance*, *d*(*A*, *B*), is the minimum number of inversions needed to transform *A *into *B*. If d is the inversion distance between *A *and *B*, then an *optimal sorting path *from *A *to *B *is a sequence of *d *+ 1 permutations *C*_0_, *C*_1_, ..., *C*_*d *_obeying *C*_0 _= *A*, *C*_*d *_= *B*, and, for all *C*_*i*_, 0 ≤ *i *<*d*, *d*(*C*_*i*_, *C*_*i*+1_) = 1. The number of such optimal sorting paths is typically very large [12].

The *(inversion) median *of three permutations *A*, *B *and *C *is a signed permutation *M *that minimizes the sum *S*(*M*) = *d*(*M, A*) + *d*(*M*, *B*) + *d*(*M*, *C*). Note that the median of three permutations need not be unique. Given permutations *A*, *B*, *C *and *X*, where *X *may or may not be a median of the first three, we call the sum *d*(*X*, *A*) + *d*(*X*, *B*) + *d*(*X*, *C*) the *(inversion) tree length *with respect to *X*; if *X *is a median, we also call the sum the *(inversion) median score *of *A*, *B*, and *C*. We write *M*_*A*, *B*, *C *_to denote the set of medians of three given permutations *A*, *B*, and *C *and *P*_*A*, *B*, *C *_to denote the set of signed permutations that lie in one or more of the optimal sorting paths between *A *and *B*, *A *and *C*, or *B *and *C*.

We call an inversion *ϕ *on permutation A *median-preserving *if we have *M*_*A.ϕ*, *B*, *C *_⊆ *M*_*A*, *B*, *C*_. Identifying median-preserving inversions could be useful in finding a median since one could then restrict a search to only such inversions. Siepel and Moret [13] first gave a characterization of such inversions.

**Theorem 1**. [13]*If permutation *X *is on an optimal sorting path from one of *A *to some median in M*_*A*, *B*, *C*_, *then M*_*X*, *B*, *C *_⊆ *M*_*A*, *B*, *C*_.

Theorem 1 does not aid in finding a median directly since it assumes that a median is already known, but, together with the triangle inequality, it can be used to derive simple bounds on the median score.

Since our new heuristic is based on a median solver for the DCJ operation, we quickly review the basic definitions for this operation. A *double-cut-and-join (DCJ) *operation makes two cuts in the genome (possibly on different chromosomes) and rejoins the resulting four cut ends in any of the three possible ways. Depending on whether the two cuts were made in the same chromosome or in two different ones and on which of the two nontrivial rejoinings is chosen, the result can be an inversion, a translocation, or a chromosomal fusion or fission. The DCJ distance, median, and median score are defined as in their counterparts for inversion, using DCJ operations in lieu of inversion operations. While DCJ is more general than inversion, its combinatorial structure is simpler [7]. However, the main algorithmic results are the same as for inversions: distance can be computed in linear time and median-finding is NP-hard.

## Results and Discussion

### Some negative results about median-finding heuristics

Searching the set *P*_*A*, *B*, *C *_for a median appears tempting; however, an inversion median cannot always be found on an optimal sorting path.

**Theorem 2**. *There exists a family of permutations: *{*A*, *B*, *C *| *M*_*A*, *B*, *C *_∩ *P*_*A*, *B*, *C *_= ∅}.

(The proof is omitted for lack of space; see additional file 1: proof.pdf)

A well known heuristic to find a median is MGR [14]. It uses so-called "good" inversions to find an approximation to the optimal median. Given permutations *A*, *B *and *C*, a *good inversion *in *A *with respect to *B *and *C *is an inversion that is both on an optimal sorting path from *A *to *B *and on an optimal sorting path from *A *to *C*. Such inversions have widely been viewed as median-preserving, justifying their use in the heuristic. Perhaps surprisingly, they are not, as can be seen by taking *A *= (1 -6 4 -3 5 -2), *B *= (3 1 -4 6 -5 2), and *C *= (-1 -5 -4 -2 -6 3), which have an optimal median score of 9. Permutation *D *= (2 -5 3 -4 6 -1) can be obtained from *A *by a single good inversion with respect to *B *and *C*, but the optimal median score of *D*, *B*, and *C *is 9, so that using a median of *D*, *B*, and *C *with *A*, *B*, and *C *yields a tree length of 10, showing that the good inversion is not median-preserving.

A more recent and better heuristic to find a median is the method of maximal signatures, originally designed for use in ancestral reconstruction [15]. A *maximal signature *of a permutation *A *with respect to permutations *B *and *C *is a permutation *D *such that there exists an optimal sorting path from *A *to *D *that consists of only good inversions (with respect to *B *and *C*) such that there is no good inversion from *D *(with respect to *B *and *C*). It is a "last permutation" that is common to sorting paths from *A *to both *B *and *C*. The following observations reveal how maximal signatures interact with the set of medians of three permutations.

**Observation 1**. *There exist three permutations A, B, and C, and a maximal signature D of A with respect to B and C together obeying M*_*D*, *B*, *C *_∩ *M*_*A*, *B*, *C *_= ∅.

In the example used for MGR, *D *is also a maximal signature and so establishes the result.

**Observation 2**. *There exist three permutations A, B, and C, a maximal signature D of A with respect to B and C, a maximal signature E of B with respect to C and A, and a maximal signature F of C with respect to A and B, together obeying M*_*D*, *E*, *F *_∩ *M*_*A*, *B*, *C *_= ∅.

As an example, take *A *= (-5 -4 1 2 3 6), *B *= (2 5 -4 -1 -3 6), *C *= (4 6 1 5 -3 2), *D *= (4 5 1 2 3 -6), *E *= (4 5 -6 3 1 2), and *F *= (-5 -1 -6 3 4 2).

These counterexamples illustrate that it is not always possible to *reduce *the problem of finding an inversion median of three given permutations to another triple of permutations obtained by good inversions from the original three permutations. Indeed, every time a good inversion is applied, it is possible to move *away *from the target set of medians.

### A framework for median-finding heuristics

Despite the fact that good inversions are not median-preserving, in practice they produce permutations with very good to near-optimal tree lengths. How the good inversions are chosen and applied, however, has a significant influence on the quality of the result. Heuristics based on good inversions differ in two parameters: the type of good inversion chosen and the method used to apply successive good inversions.

The good inversion can be chosen at random from the set of all possible good inversions. A better choice is to select a good inversion that, once applied, leaves as large a set of available good inversions (for the next permutation) as possible; we call such a choice a *greedy good inversion*. On a permutation of length *n*, all sorting inversions can be found in *O*(*n*^3^) time. To find a good inversion, sorting inversions must be found with respect to two permutations and their intersection can be computed in *O*(*n*^2^) time. To find a greedy good inversion, all good inversions must be counted for each permutation that can be obtained by using a good inversion on the current permutation. Thus finding a greedy good inversion takes *O*(*n*^5^) time.

The different methods used to apply good inversions can be thought of as parallel vs. serial, and stepwise vs. groupwise (signature).

**Figure 1:**
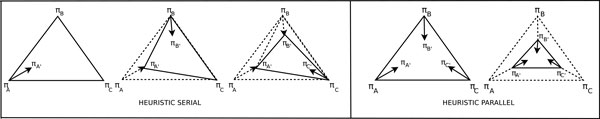
**Heuristics Serial and Parallel**. A schematic of Heuristic Serial and Heuristic Parallel.

**Heuristic 'Serial' (HS): **Use a good inversion on *A *with respect to *B *and *C *to get *A*^1^, then a good inversion on *B *with respect to **A**^1 ^and **C **to get *B*^1 ^and then a good inversion on *C *with respect to **A**^1 ^and **B**^1 ^to get *C*^1 ^(see Fig. 1). Continue iteratively (on *A*^1^, *B*^1 ^and *C*^1 ^and so on) until no good inversions are possible from *any one *of the permutations. Let the final permutations be *A*^*n*^, *B*^*n *^and *C*^*n*^. Output that of the three with the best tree length. **Heuristic 'Serial Extended' (HSE) **is the same as Heuristic HS except for the stopping condition. The iterative process stops when no good inversions are possible from *all *three permutations.

**Heuristic 'Parallel' (HP): **Use a good inversion on *A *with respect to *B *and *C *to get *A*^1^, then a good inversion on *B *with respect to **A **and **C **to get *B*^1 ^and then a good inversion on *C *with respect to **A **and **B **to get *C*^1 ^(see Fig. 1). Continue iteratively (on *A*^1^, *B*^1 ^and *C*^1 ^and so on) until no good inversions are possible from *any one *of the permutations. Let the final permutations be *A*^*n*^, *B*^*n *^and *C*^*n*^. Output that of the three with the best tree length. **Heuristic 'Parallel Extended' (HPE) **is the same as Heuristic HP except for the stopping condition. The iterative process stops when no good inversions are possible from *all *three permutations.

**Figure 2:**
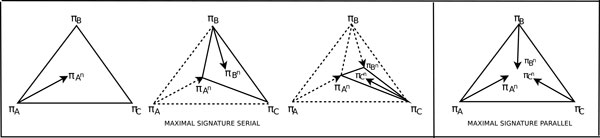
**Maximal Signature heuristics**. A schematic of Heuristic Maximal Signature Serial and Heuristic MAximal Signature Parallel.

**Heuristic 'Maximal Signature Serial' (MSS): **Find a maximal signature of *A *with respect to *B *and *C*: *A*^*n*^, a maximal signature of *B *with respect to **A**^**n **^and **C**: *B*^*n *^and a maximal signature of *C *with respect to **B**^**n**^and **A**^**n**^: *C*^*n *^(see Fig. 2). Output that of *A*^*n*^, *B*^*n*^, and *C*^*n *^with the best tree length.

**Heuristic 'Maximal Signature Parallel' (MSP): **Find a maximal signature of *A *with respect to *B *and *C*: *A*^*n*^, a maximal signature of *B *with respect to **A **and **C**: *B*^*n *^and a maximal signature of *C *with respect to **B **and **A**: *C*^*n *^(see Fig. 2). Output that of *A*^*n*^, *B*^*n*^, and *C*^*n *^with the best tree length and call it the *Maximal Signature Median (MSM)*.

Heuristic Serial changes the permutations on which the computation of good inversions is based at every step, while heuristic Parallel changes it at every three steps. MGR-Median uses one of these two heuristics (which one is not clear from the literature) with greedy good inversions, followed by a local search to reduce the tree length. The Heuristic Maximal-Signature-Serial makes the corresponding changes only twice in the entire computation, whereas Maximal-Signature-Parallel never changes the permutations with respect to which good moves are computed. In this sense, these heuristics are arranged in descending order of how often the problem is reduced to finding a median on a new triple of permutations.

### A new heuristic for the inversion median

While MSM does very well in practice, it remains too slow (at least in a naïve implementation) to use on genomes with tens of thousands of genes. We therefore set out to design a new heuristic that would never change the data from which new values are computed, would be at least as accurate as MSM, but would also run fast enough to enable the handling of mammalian-size gene orders. As mentioned earlier, computing with the DCJ operation has been found to be somewhat simpler and more efficient than computing with the inversion operation, so we turned to DCJ medians as a way to obtain a fast approximation to an inversion median--a reasonable approach, as DCJ operations reduce to inversions if splitting chromosomes is not allowed. Previous work showed that inversion and DCJ distances remain very close for pairs of unichromosomal genomes under a variety of evolutionary scenarios [16], so it also appears likely that DCJ medians and inversions medians are closely related.

Because DCJ operations can split a chromosome into two smaller chromosomes, the optimal DCJ median of three unichromosomal genomes could be a multichromosomal genome. Forcing the median to be unichromosomal makes median computations much more expensive. Therefore, we consider the "relaxed" version of the problem, in which the median produced by the DCJ median solver may contain extra (small) chromosomes; if such extra chromosomes exist, we handle them separately, using a few DCJ operations to merge them into the main chromosome. Clearly, good performance will depend on there being very few of these extra chromosomes.

Finding an optimal DCJ median is, like finding an optimal inversion median, an NP-hard problem [8,9], so we base our approach on designing a good heuristic for the DCJ median problem, using a relaxation of the optimal DCJ median solver developed by one of us [17,18]. That solver relies on the notion of *adequate subgraphs *to decompose a full instance into smaller instances, a process that is repeated until a solution to the original instance is found. The decomposition itself preserves optimality and does not affect the input data--in that sense, the optimal median solver obeys our main recommendation for median heuristics. Adequate subgraphs, however, can have any size and finding large adequate subgraphs is hard; in practice, the solver uses a fixed collection of small adequate subgraphs, the presence of which can be detected in linear time. If none of the adequate subgraphs in the collection is detected, the solver resorts to exhaustive search--and it is at this level that a heuristic for the DCJ median problem must be designed.

The DCJ median problem can be modelled with a *multiple breakpoint graph (MBG)*, a regular graph of degree equal to the number of given genomes, in which each adjacency between two genes in one genome is represented by a colored edge, using one color for each genome. Edges representing adjacencies from a pair of genomes thus form a collection of color-alternating cycles. Under the DCJ operation, finding a median is equivalent to creating a perfect matching to form the maximum number of color-alternating cycles with the existing edges on the MBG [8,17]. Adequate subgraphs in effect choose certain edges to place in this matching. In designing a heuristic, we must then consider how to select the next edge to add to the current (incomplete) matching.

An obvious choice for selecting an edge is to pick that edge whose addition to the matching results in the largest number of color-alternating cycles. A less obvious criterion is to match (part of) the definition of an adequate subgraph--that way, the edge chosen may in fact implicitly represent an adequate subgraph, which we know is a good strategy. The critical quantity in the definition of an adequate subgraph is the *adequacy*, defined as , where *l *is the number of edges and c the number of cycles [17]. So a second criterion is to choose that edge which maximizes the adequacy of the solution built so far.

The complementary question for selection is what to search; if the current matching requires *m *additional edges, then there are 2*m *unmatched vertices left and  possible edges to choose from. Evaluating each such edge may prove expensive, since the growth is quadratic; however, we are designing a heuristic, so we can arbitrarily restrict the search in order to speed up this step, e.g., by using a vertex ordering and focusing on the next vertex only, thereby cutting down the number of potential edges to 2*m *- 2, or further restricting the choice to just the three existing edges incident upon that vertex, a constant. In all three cases, after adding the new edge to the matching and removing the two matched vertices, we resume the overall procedure, that is, we resume searching for adequate subgraphs in the remaining graph. The running time of the complete heuristic is thus quartic for the search among all potential edges, cubic for the search focused on the next vertex alone, and quadratic for the search restricted to the three existing incident edges, giving us a range of tradeoffs between speed and accuracy; we call these three versions *ASM4*, *ASM3*, and *ASM2 *respectively. Finally, once the approximate median is produced, we use a greedy procedure to merge any extra circular chromosomes into the main chromosome: a merging DCJ operation that minimally increases the total DCJ distance is used, and the step is repeated until no extra chromosome remains.

From the results of extensive experimentation over a large range of genome sizes (not shown), we chose to use the adequacy criterion rather than the cycle criterion for evaluating a potential new edge. The choice of search strategy depends in part on the value attached to very high speeds, but we chose to show data obtained with the slowest of the three variants, ASM4, mostly because it is the most accurate of the three and yet is orders of magnitude faster than its closest competition.

### Experimental results

We conducted extensive experiments for all of the heuristics described, including MGR, every heuristic in the framework, and ASM, on genomes of small sizes (up to 200 genes, a size that covers organelles) and of large to very large sizes (from 1'000 to 25'000 genes), the latter focused on ASM, as it is clearly the best of the heuristics in terms of both accuracy and running time. Some of our genomes are generated as random permutations, while others are generated from a known ancestor with controlled numbers of inversions. Details of the experimental setup can be found under Methods.

We present and discuss the results in several groups: those for small genomes and heuristics based on good inversions, which we conducted to gain insight into the classification framework and test our conjecture (that the heuristic that does not deviate from computation with respect to the original three permutations outperforms the others), as well as to verify that ASM would indeed dominate all such heuristics; those for genomes evolved through controlled numbers of inversions, which we conducted to evaluate the effect of edge lengths on the heuristics (and incidentally to observe the difference between ancestors and medians); and those for large genomes, in which we evaluate ASM on its own.

#### Heuristics based on good inversions

Table 1 shows the average tree lengths (averaged over 1'000 triples of random permutations) for all heuristics based on good inversions using greedy good inversions. (Other results, not shown, indicate that greedy good inversions always do better than random good inversions.) It also shows the average scores for MGR and for "trivial medians" (the best of the three original genomes used in lieu of a median) computed by MGR for the same sets of permutations. The standard deviation for heuristic MSP varies from 1.3 to 1.6 and that for heuristic HPE (that with the largest standard deviation) varies from 1.7 to 2.3. The change in stopping condition in heuristics HS and HP has no significant impact on the performance and all four versions perform on par with MGR. The latter is both to be expected (since MGR uses one of these four in its first stage) and somewhat disappointing, as it indicates that the expensive local search run by MGR in its second stage has little effect on the score. Overall, heuristic MSP gives clearly better results than the other heuristics; moreover, the difference in median score between MSP and other heuristics is always non-negative, making MSP the best-scoring heuristic in every test case, not just on average.

**Table 1:** Comparison of (greedy) good inversion based heuristics. Average tree length for random permutations (MGR-H1 used for length 100).

Length	MSP	MSS	HS	HSE	HP	HPE	MGR	Trivial
30	46.62	47.31	48.68	48.01	49	48.16	47.23	55.8

50	80.31	81.29	84.11	83.23	84.31	83.5	82.01	94.99

100	167.27	168.48	174.49	173.42	174.56	173.75	181.54	194.35

These experiments confirm our conjecture based on the framework presented above: the best heuristic is that which deviates the least from computation with respect to the original three permutations. While computing with original data is unusual in the realm of greedy heuristics, it makes perfect sense in this context. Consider *S*, the intersection of the sorting paths *P*(*A*, *B*) and *P*(*A*, *C*), and S', the intersection of the sorting paths *P*(*A*, *B*') and *P*(*A*, *C'*), where *B' *(resp., *C'*) is one good inversion away from *B *(resp., *C*) with respect to *A *and *C *(resp., *A *and *B*). Since the distance between *A *and *B' *(resp., *C'*) is one less than the distance between *A *and *B *(resp., *C*), the number of permutations in *S' *is lesser than the number of permutations in *S*--indeed, we have *S' *⊆ *S*. In the Serial and Parallel heuristics, the search space reduces at each step, whereas in the heuristics based on maximal signatures, a larger space (a strict superset) is searched at every step, leading to the better quality of the scores.

#### ASM, MGR, and MSM on random permutations

**Figure 3:**
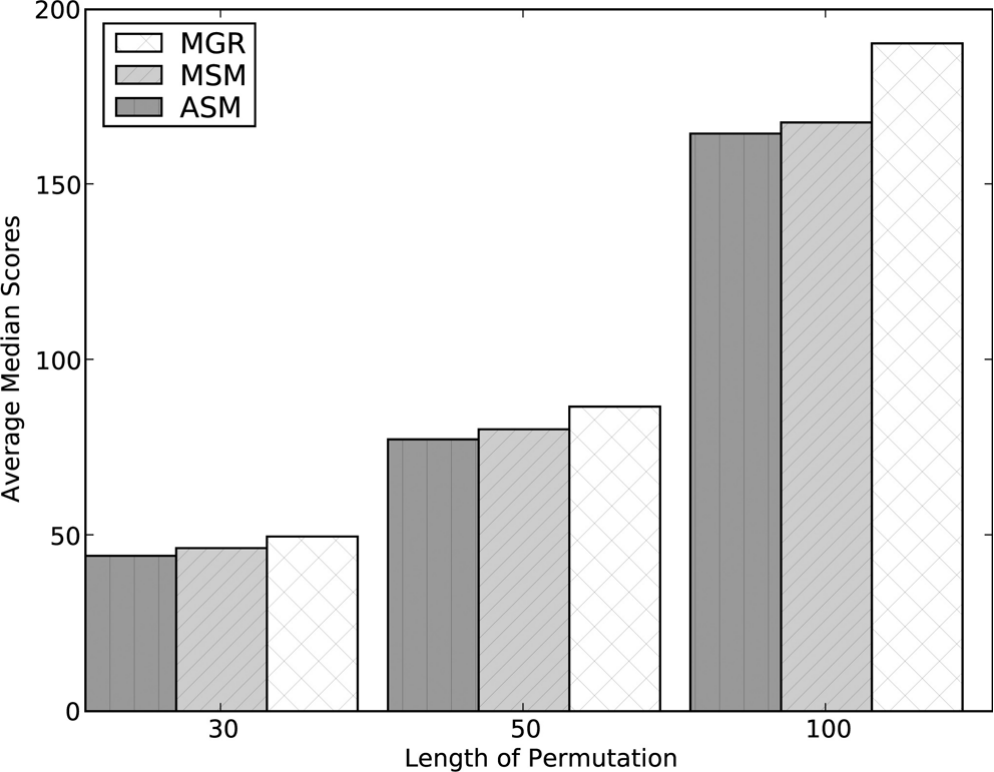
**Performance of median solvers MGR, MSM and ASM on Random Permutations: average tree length**. Average tree length on random permutations (MGR-H1 used for length 100).

**Figure 4:**
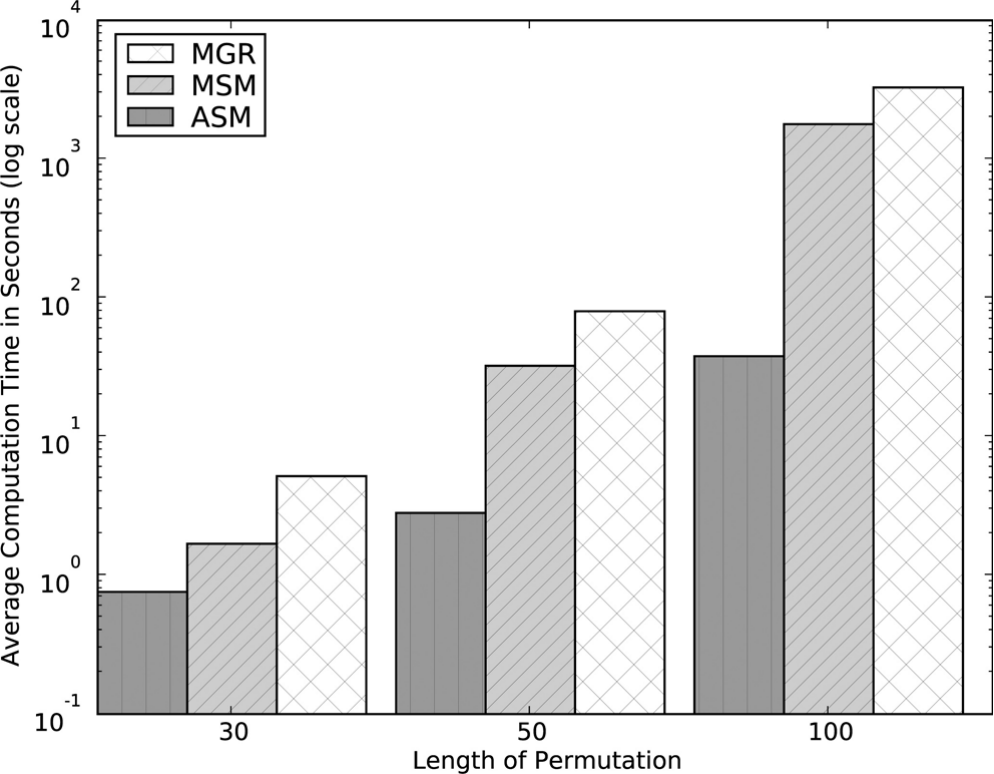
**Performance of median solvers MGR, MSM and ASM on Random Permutations: average running time**. Average running time on random permutations (MGR-H1 used for length 100).

Figs. 3 and 4 show a comparison (averaged over 100 random triples of permutations) of the three solvers of highest interest, ASM4, MGR, and MSM in terms of tree lengths and of running times respectively. ASM4 and MSM consistently outperform MGR in both accuracy and speed. ASM4 is clearly the fastest of the three and also slightly outperforms MSM in accuracy.

#### ASM, MGR, and MSM under simulation

**Figure 5:**
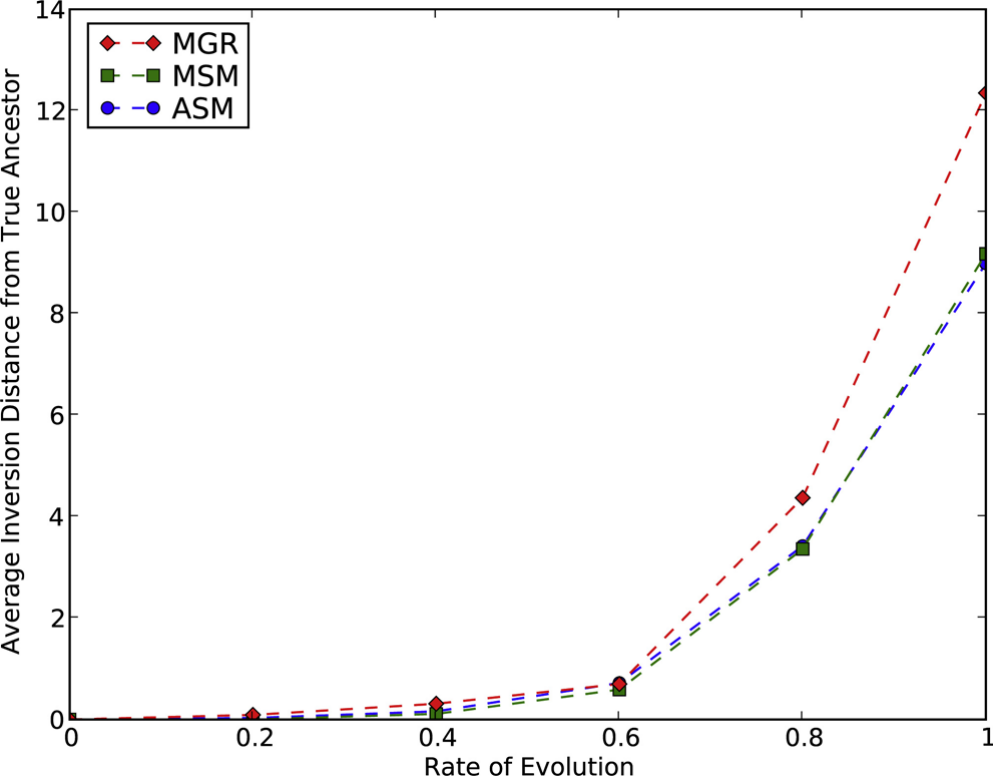
**Ancestral reconstruction: MGR, MSM and ASM under simulation (inversions only)**. Inversion distance from the approximate median to the true ancestor on permutations generated through k inversions from the ancestral permutation.

**Figure 6:**
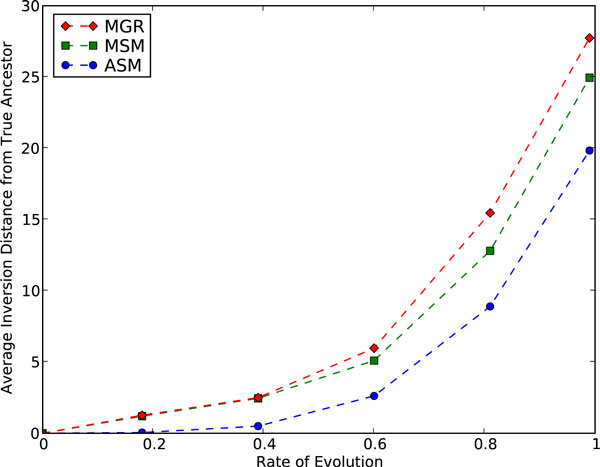
**Ancestral reconstruction: MGR, MSM and ASM under simulation (inversions and transpositions)**. Inversion distance from the approximate median to the true ancestor on permutations generated through 0.8 k inversions and 0.2 k transpositions from the ancestral permutation.

We also conducted simulations to compare ASM4, MGR, and MSM. Maximal signatures have been used previously for ancestral construction [15], but not through a median-based approach. We ran tests on both symmetric and asymmetric 3-leaf trees and found that symmetric trees generated harder instances, in terms of both accuracy and running time, so we present results only for symmetric trees. The average inversion distance between the identity (true ancestor) and the approximate medians computed by the three median solvers, under a regime of inversions only is shown is Fig. 5 and under a mix of inversions and transpositions is shown in Fig. 6, the latter used to test sensitivity to model choice. These tests show that ASM4 matches the performance of MSM, the best ancestral reconstruction tool to date, on inversion-only scenarios, and does better for mixed scenarios, presumably a consequence of its DCJ origins. Ancestral reconstruction is not our main focus in this paper, but median solvers are usually the tool of first recourse for such reconstruction, and ASM is quite promising in this regard as well.

**Figure 7:**
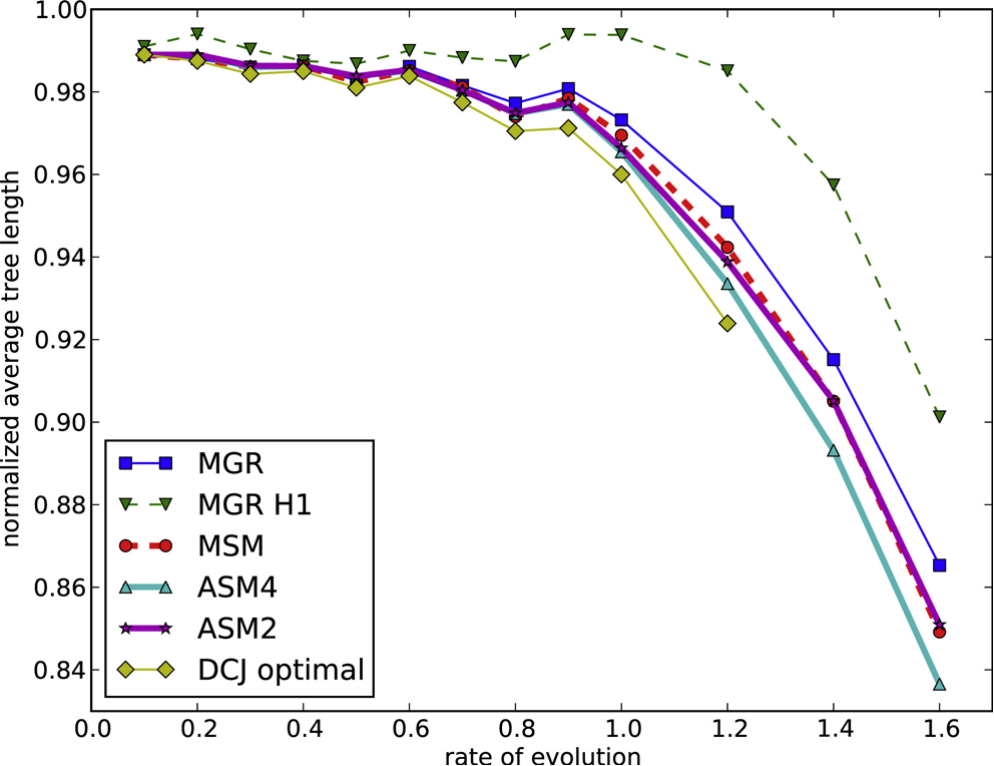
**Performance of various median solvers on genomes of size 100: average tree length**. Average tree length on genomes of size 100.

**Figure 8:**
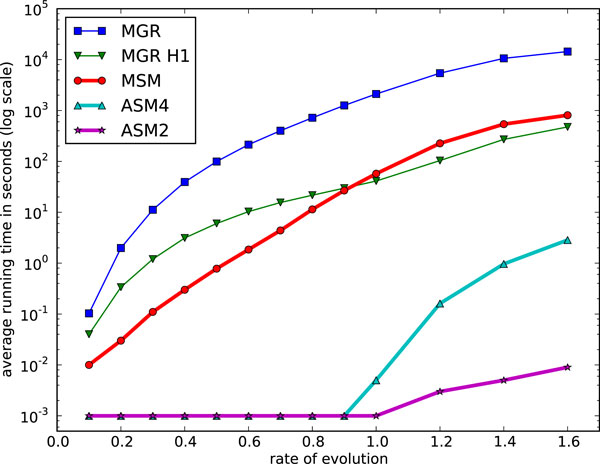
**Performance of various median solvers on genomes of size 100: average running time**. Average running time on genomes of size 100.

**Figure 9:**
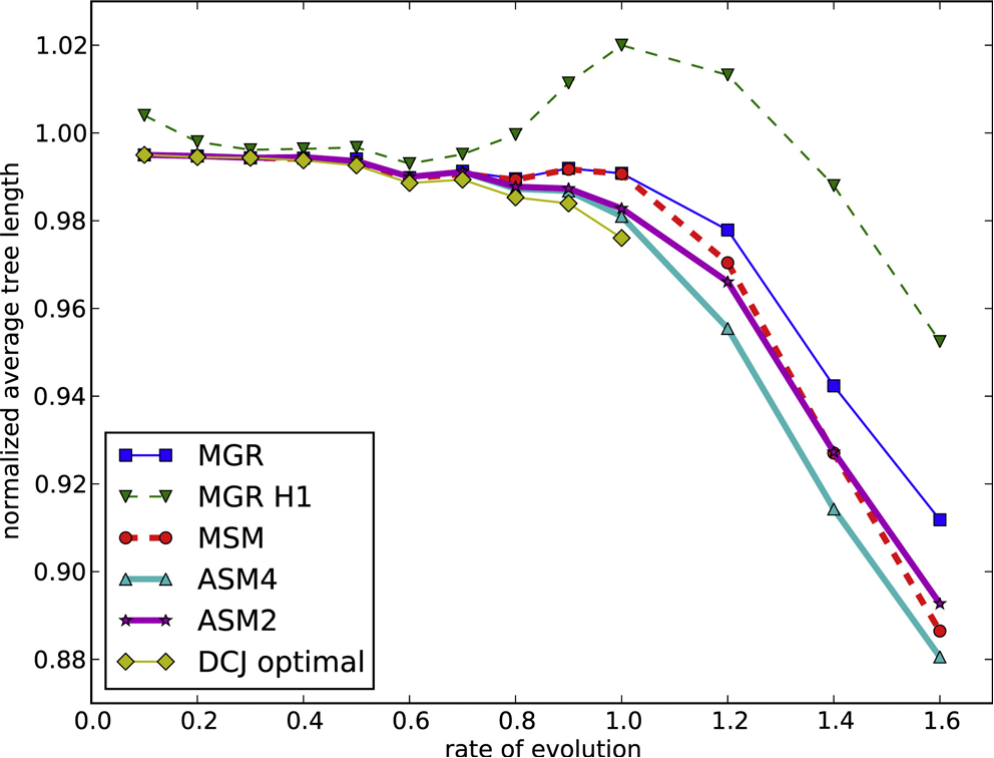
**Performance of various median solvers on genomes of size 200: average tree length**. Average tree length on genomes of size 200.

**Figure 10:**
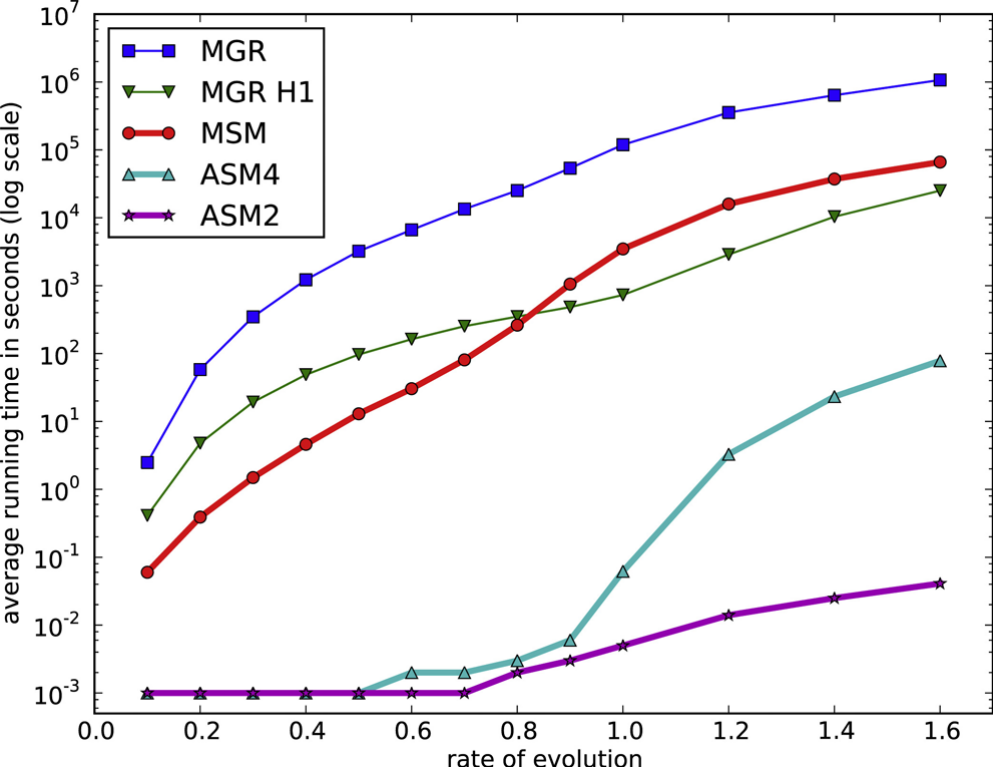
**Performance of various median solvers on genomes of size 200: average running time**. Average running time on genomes of size 200.

We expanded these simulation tests to genomes of 100 and 200 genes, and then to large genomes of 1'000, then 5'000, then finally 25'000 genes. The last three match the size of data generated by syntenic block analyses (1'000), large prokaryotic or small eukaryotic genomes (5'000), or mammalian genomes (25'000). We used a total number of inversions varying from 0.1*n *to 1.6*n *(to 0.9*n *only for the largest genomes), where *n *is the number of genes. Figs. 7 and 8 show the results (average scores and average running times respectively) for genomes of 100 genes. The same results for genomes of 200 genes are shown in Figs. 9 and 10. We give results for both MGR and MGR-H1 (MGR run with the weaker H1 option). We also give the DCJ median score, which provides a lower bound on the inversion median score. The ASM4 tree length tracks very closely the DCJ median score. Up to a rate of 0.8, the two are indistinguishable, indicating that ASM4 returned true inversion medians. Beyond that point, the two differ slightly, but the difference remains very small. Moreover, the heuristics are totally ordered, with ASM4 dominating MSM, MSM dominating MGR, and MGR dominating MGR-H1.

Turning to running times, the difference between ASM4 and the other heuristics is much more pronounced. Not only is ASM4 very fast, but it is nearly unaffected by the evolutionary rate, up to 0.9, whereas all others slow down quickly as the tree lengths increase--it should be noted that the time scale is logarithmic. At *r *= 0.9, ASM4 still takes milliseconds, whereas MSM takes minutes and MGR takes hours. Beyond that point, ASM4 slows down quickly, but even at *r *= 1.6 (a very large evolutionary rate, since rearrangements are rare events), it runs in a few minutes, whereas MSM now takes hours and MGR takes days. If more speed is desirable, ASM2 can be used: it still runs in milliseconds at *r *= 1.6, at the expense of some accuracy.

**Figure 11:**
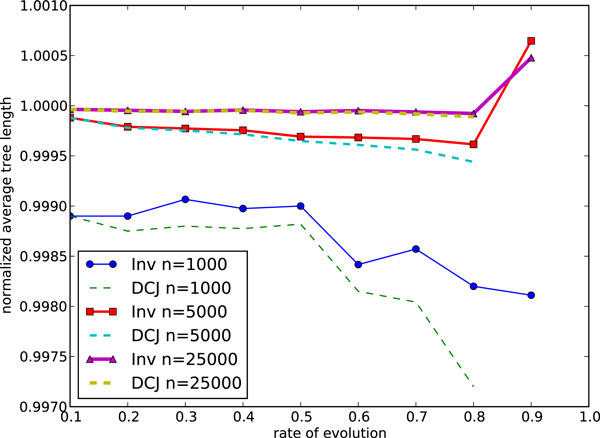
**Performance of ASM4 on very large genomes: average tree length**. Average tree length on large genomes.

**Figure 12:**
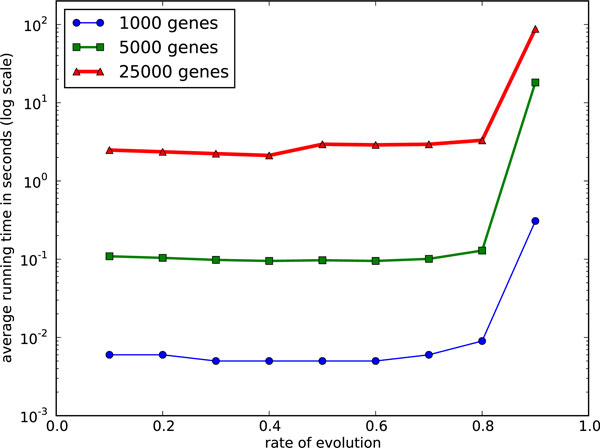
**Performance of ASM4 on very large genomes: average running time**. Average running time on large genomes.

Scaling to 1'000 genes goes beyond the capabilities of MGR-H1 and scaling to 10'000 genes goes beyond the capabilities of MSM, so our scaling study focuses exclusively on ASM4, using a restricted evolutionary range from 0.1 to 0.9. Figs. 11 and 12 show the DCJ median scores and average tree lengths given by ASM4 and the average running times for ASM4 respectively for the three large genome sizes. As the number of genes grows, the running time naturally increases, but note that, for *r *≤ 0.8, ASM4 takes only a few seconds on genomes of 25'000 genes. As for accuracy, the DCJ lower bound indicates that, the larger the genome, the more accurate (for a constant evolutionary rate *r*) the answer returned by ASM4 is. Indeed, the solution is optimal for most instances of 25'000 genes and *r *≤ 0.8. Thus computing an approximate median for full gene orders (under our initial simplifying assumptions) is no longer an issue: it can be done very quickly and very accurately up to large evolutionary distances.

For a final perspective, we tabulated the number of additional small circular chromosomes placed in the solution returned by ASM4. As expected in view of the success of the heuristic, Table 2 shows that this number is very small, usually below one, except for the largest genomes at the highest evolutionary rates.

**Table 2:** Extra circular chromosomes found in ASM4 solution. The average number of extra circular chromosomes in the DCJ median found by ASM4.

	rate of evolution								
**# genes**	**0.1**	**0.2**	**0.3**	**0.4**	**0.5**	**0.6**	**0.7**	**0.8**	**0.9**

100	0.00	0.01	0.04	0.05	0.21	0.30	0.36	0.64	0.81

200	0.00	0.00	0.03	0.08	0.26	0.25	0.39	0.79	0.95

1'000	0.00	0.01	0.04	0.11	0.16	0.31	0.57	0.97	1.33

5'000	0.00	0.01	0.05	0.12	0.17	0.32	0.54	0.97	1.75

25'000	0.01	0.02	0.02	0.10	0.17	0.35	0.49	0.81	1.88

## Conclusion

We have presented a unifying framework in which to classify and analyze greedy heuristics for the inversion median problem. We gave counterexamples to a number of reasonable assertions about greedy inversions and the relationships between inversion medians and inversion sorting paths among the three given genomes. We used insights derived from our framework to conclude that the best heuristics continue to base their decisions on the original input data rather than on refined estimates and showed that such a strategy maximizes the number of useful choices available at each step. We gave a new heuristic that follows this principle, but operates in the space of DCJ operations rather than inversion operations. Finally, we presented extensive experimental results supporting our conclusions about good inversion-based heuristics and showing that our new DCJ-based heuristic, ASM, clearly dominates all existing heuristics in terms of both accuracy and running time. In particular, we showed that ASM can handle full mammalian gene orders (25'000 genes) in seconds and, even for considerable evolutionary distances, return a solution that is optimal or very nearly so. As biological data is of limited size for any given genome, ASM4 appears sufficient to the task of analyzing the rearrangement history of even the biggest genomes. Its remaining limitations are also those of every solution proposed so far: it cannot deal directly with duplications and losses, nor can it handle additional constraints (such as constraints on the length of inversions). Further work will thus include the refinement of ASM to handle gene families under simple models of duplication and loss, along with applications to biological data drawn from the Tree of Life that will help identify relevant biological constraints on inversions.

## Methods

Our first set of experiments uses random permutations of lengths 30, 50, and 100 and are used to compare heuristics based on good inversions and to yield a first comparison of MSM, MGR and ASM4. Each dataset consists of three randomly generated permutations. The version of MGR used is 2.01, with the -*c *option (condensing strips for efficiency) used in all cases, and the -*H1 *option used for permutations of length 100 (so as to speed up the computation--otherwise MGR often takes several days to compute the scores).

All other experiments are based on simulated evolution. A dataset is a 3-leaf tree, with the identity permutation placed at the internal node and a permutation at a leaf derived from the identity by applying l random inversions (in one case, as noted in the text, a mix of inversions and transpositions), where l is the length of the edge from the internal node to the leaf. We generate symmetric trees, in which all three edges have the same length, although we also use (data not shown) asymmetric trees where one edge has twice or thrice the length of the other two. We generally give the sum of the three edge lengths as the total evolutionary distance; we also give the same information as the evolutionary rate for the tree, where the evolutionary rate, *r*, is simply the total evolutionary distance, *l*, divided by the size of the permutations, *n*. These tests are used to compare ASM4, MGR, MGR-H1, and MSM, plus running times on the largest datasets for ASM4. Once again, MGR is version 2.01 used with the -*c *option and MGR-H1 is MGR with both the -*c *and -*H1 *options. The DCJ median scores (optimal values) for these datasets are computed with the exact solver of Xu and Sankoff [17].

## Competing interests

The authors declare that they have no competing interests.

## Authors' contributions

VR constructed the counterexamples for good inversions, conceived the classification of heuristics, and conjectured that the use of original values would outperform the use of updated values. AWX conceived and developed ASM, the DCJ-based heuristics. VR and AWX implemented all heuristics and conducted the experimental testing. VR, YL and KMS proved theorem 2. BMEM directed the project. Finally, VR, AWX and BMEM collaborated closely on the experimental protocol, the analysis of the results, and the writing of the manuscript.

## Supplementary Material

Additional file 1 **Proof of Theorem 2**. contains the proof of Theorem 2. Click here for file
