# Supplementary material for: Heuristics for the inversion median problem
Source: BMC Bioinformatics. 2010 Jan 18;11(Suppl 1):S30. doi: 10.1186/1471-2105-11-S1-S30 (PMC3009502; doi:10.1186/1471-2105-11-S1-S30)
Supplement: Additional file 1 — Proof of Theorem 2. contains the proof of Theorem 2. [file 1471-2105-11-S1-S30-S1.pdf]

Proof of Theorem 2

Notation: For signed permutations A and B, we denote the *inversion distance* by $d(A,B)$. Given permutations A, B, C and X, where X may or may not be a median of the first three, we call the sum $d(X,A)+d(X,B)+d(X,C)$ the *tree length* with respect to X; if X is a median, we also call the sum the *median score* of A, B, and C.
We write $M_{A,B,C}$ to denote the set of inversion medians of three given permutations A, B, and C and $P_{A,B,C}$ to denote the set of signed permutations that lie in one or more of the optimal (inversion) sorting paths between A and B, A and C, or B and C.
We denote by $P(A,B)$ the set of all permutations that lie on sorting paths from A to B. Given permutation x_{12}, that lies on a sorting path from A to B, such that x_{12} is closest to C we call $d(C,x_{12})$ the *closest-path distance* of C.

Theorem 2. *There exists a family of permutations:* $\{A,B,C \mid M_{A,B,C} \cap P_{A,B,C} = \emptyset\}$.

Proof. Let $\phi_1 = (4\,3\,1\,2)$, $\phi_2 = (4\,2\,1\,3)$ and $\phi_3 = \iota$, the identity. The following facts can be easily checked from the sorting paths of these permutations:

1. $d(\phi_1,\phi_3) = 3$, $d(\phi_1,\phi_2) = 2$, $d(\phi_2,\phi_3) = 2$.
2. The permutation in $P(\phi_1,\phi_2)$ that is closest to ϕ_3 is ϕ_2 so ϕ_3 has closest-path distance 2.
3. The permutation in $P(\phi_2,\phi_3)$ that is closest to ϕ_1 is ϕ_2 so ϕ_1 has closest-path distance 2.
4. The permutations in $P(\phi_3,\phi_1)$ that are closest to ϕ_2 are ϕ_1 and ϕ_3, so ϕ_2 has closest-path distance 2.
5. The tree length of ϕ_2 with respect to ϕ_1, ϕ_2 and ϕ_3 is 4.
6. The tree length of ϕ_3 and ϕ_1 are both 5; hence neither is a median.

Let $C1$, $C2$ and $C3$ be the breakpoint graphs of ϕ_1, ϕ_2 and ϕ_3 respectively. We construct A in such a way that its breakpoint graph is a concatenation of $C3$, $C1$ and $C2$ (in that order). Similarly, B is a concatenation of $C1$, $C2$ and $C3$ and C is a concatenation of $C2$, $C3$ and $C1$. Thus,

$A = (1\,2\,3\,4\,\mathbf{5}\,9\,8\,6\,7\,\mathbf{10}\,14\,12\,11\,13)$,
$B = (4\,3\,1\,2\,\mathbf{5}\,9\,7\,6\,8\,\mathbf{10}\,11\,12\,13\,14)$, and
$C = (4\,2\,1\,3\,\mathbf{5}\,6\,7\,8\,9\,\mathbf{10}\,14\,13\,11\,12)$.

The permutations have two extra elements (**5** and **10**) to separate the three components from each other. The distances are: $d(A,B) = d(B,C) = d(C,A) = 7$.
Consider the permutation constructed by concatenating $C2$ three times: $m = (4\,2\,1\,3\,\mathbf{5}\,9\,7\,6\,8\,\mathbf{10}\,14\,12\,11\,13)$. Permutation m does not lie on any of the sorting paths amongst A, B and C because the component $C2$ is never on a sorting path from $C1$ to $C3$. Note that $d(A,m) = d(B,m) = d(C,m) = 4$ and so, the tree length of m is 12. We now show that there is no permutation p in $P(A,B)$ that is closest to C with tree length as small as 12 (in other words, p cannot be a median). Since components are

sorted independently (in the absence of hurdles), we now look only at the first component of p ($C1_p$). Consider a permutation on the sorting path from the first component of A ($C3$) to the first component of B ($C1$) that is closest to the first component of C ($C2$). The closest-path distance of the first component of C ($C2$) with respect to $C1_p$ is 2: from fact 4. Thus, the minimum score for a permutation from $P(C3, C1)$ is obtained by choosing $C3$ or $C1$ as $C1_p$, contributing 5 $(= d(\phi_1, \phi_3) + d(\phi_1, \phi_2))$ to the total tree length. If we choose $C1_p$ such that it is not on a path from $C3$ to $C1$ we can take $C1_p = C2$ which yields a score of 4. But then the same situation occurs when limited to only the second components or only the third components; no matter which set we consider—$P(A,B)$, $P(B,C)$, or $P(C,A)$—we are forced to choose a component for p that is on a path from $C3$ to $C1$. Thus, one component will have score of at least 5 while the other two will have a score of at least 4, yielding a total tree length not lesser than 13.

Due to the fact that components are sorted individually, we can build a permutation of any length n with the same properties by simply appending any permutation of length $n - 14$ (renamed appropriately) to the end of this example.
